# Spinal dermoid sinus in a Dachshund with vertebral and thoracic limb malformations

**DOI:** 10.1186/1746-6148-10-54

**Published:** 2014-03-04

**Authors:** Natasha Barrios, Marcelo Gómez, Marcelo Mieres, Frank Vera, Genaro Alvial

**Affiliations:** 1 Hospital Veterinario, Universidad San Sebastián, Puerto Montt, Chile; 2 Instituto de Farmacología y Morfofisiología, Universidad Austral de Chile, Casilla 567, Valdivia, Chile; 3 Instituto de Ciencias Clínicas Veterinarias, Universidad Austral de Chile, Casilla 567, Valdivia, Chile; 4 Institutio de Anatomía, Histología y Patología, Universidad Austral de Chile, Casilla 567, Valdivia, Chile

**Keywords:** Dermoid sinus, Klippel-Feil syndrome, Limbs malformations, Dog

## Abstract

**Background:**

Dermoid sinus is an uncommon epithelial-lined fistula that may be associated with vertebral malformations. In humans, Klippel-Feil syndrome (KFS) is a rare condition characterized by congenital cervical vertebral fusion and may be associated with other developmental defects, including dermoid sinus. The present case report describes an adult Dachshund with cervical and cranial thoracic vertebral malformations as well as thoracic limb malformations resembling KFS with a concurrent type IV dermoid sinus.

**Case presentation:**

A 1.5 year-old Dachshund with congenital thoracic limbs deformities and cervical-thoracic vertebral malformations presented with cervical hyperesthesia, rigidity of the cervical musculature and tetraparesis. Neurologic, radiographic, and computed tomography (CT) (2D, 3D, CT fistulography) examinations revealed skeletal anomalies, a dermoid sinus in the cranial thoracic region and epidural gas within the vertebral canal. Surgical resection and histopathological evaluation of the sinus tract were performed and confirmed a type IV dermoid sinus. The clinical signs progressively recovered postoperatively, and no recurrent signs were observed after 6 months of follow-up.

**Conclusions:**

Cervical vertebral malformations associated with limbs anomalies have not been reported in dogs and may represent a condition similar to KFS in humans. KFS can occur concurrently with other congenital conditions including dermoid sinus and should be included among the complex congenital anomalies described in dogs.

## Background

Dermoid sinus (dermoid sinus tract, dermoid cyst, pilonidal sinus, or pilonidal cyst) is an uncommon developmental defect characterized by failed separation of the ectoderm and neuroectoderm during the early embryonic stage [1-3]. Dermoid sinus is generally benign: it occurs at the dorsal midline and connects the skin to deeper spinal structures, including the vertebral canal [1-4]. Spinal dermoid sinus occur as single or multiple lesions in the cervical, cranial thoracic, thoracolumbar, lumbosacral, and sacrococcygeal regions [5-9].

**Figure 1:**
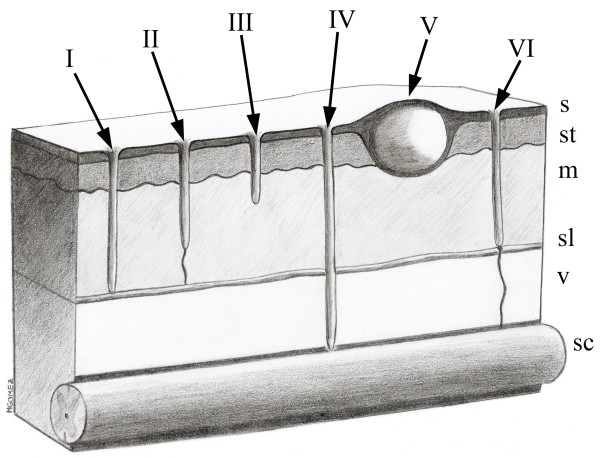
**Schematic illustration of the six types (I to VI) of dermoid sinus; s: skin; st: subcutaneous tissue; m: epaxial musculature; sl: supraspinous ligament; v: vertebrae; sc: spinal cord and meninges into the vertebral canal.** (Illustrated by Marcelo Gómez).

There are six types of dermoid sinus described in veterinary medicine categorized by the relationship between the sinus and supraspinous ligament, and the presence of a skin orifice (Figure 1) [4,6,7,10]. Type I dermoid sinus is a tubular sac extending from the skin to the supraspinous or nuchal ligament. Type II is characterized by a more superficial tract than type I and connects to the supraspinous ligament by a fibrous strand. Type III is a sac that ends proximal to the supraspinous ligament and is not connected to it by a fibrous strand. Type IV is a deeper tract that communicates with the vertebral canal and is attached to the dura mater. Type V is considered a true dermoid cyst because it is a closed sac or capsule with no connection to the skin surface, and it can be located anywhere between the skin and the vertebrae [7,10]. In a newly suggested Type VI dermoid sinus, there is an open sinus tract that extends about to the level of the supraspinous ligament but has a distal connecting strand with the dura mater [6]. Dermoid sinus is also classified into subtypes a, b, and c, depending on whether the lesion occurs in the vertebral canal, cranium or nose, respectively [11]. Neurological signs are uncommon in affected animals and usually occur in types IV or VI because of the communication between the sinus and the dura mater, which enables infections such as abscessation, meningitis, meningomyelitis or myelitis [6-8,12-14]. Additionally, spinal cord compression may develop secondary to an enlarged distal sinus tract accumulating debris such as hair follicles, sebum and keratin debris or desquamated epithelial cells [8,14]. Tethering of the spinal cord and syringomyelia associated with dermoid sinus have also been reported in dogs [6]. Neurological signs vary and depend on the neuroanatomical location of the dermoid sinus.

Craniovertebral or vertebral malformations (e.g. spina bifida, spinous process malformations, hemivertebrae, and block vertebrae) may be associated with congenital intracranial or spinal dermoid sinus [6,12,14-18]. Vertebral anomalies associated with limbs malformations are rare in small animals [19,20]. In humans, congenital fusion and shortened cervical vertebrae (cervical synostosis) known as Klippel-Feil syndrome (KFS) is a congenital anomaly equivalent to block vertebrae observed in companion animals [21-24]. Klippel-Feil syndrome is caused by failed cervical somite segmentation during embryogenesis [23]. However, KFS is usually associated with other multiple systemic anomalies such as scoliosis, visceral defects, deafness, and thoracic anomalies [21]. This complex disorder may be observed concurrently with dermoid sinus and other neurological anomalies in humans. The present short communication describes an adult dog diagnosed with a type IV dermoid sinus, subtype a with axial and appendicular malformations, resembling a Klippel-Feil syndrome.

## Case presentation

**Figure 2:**
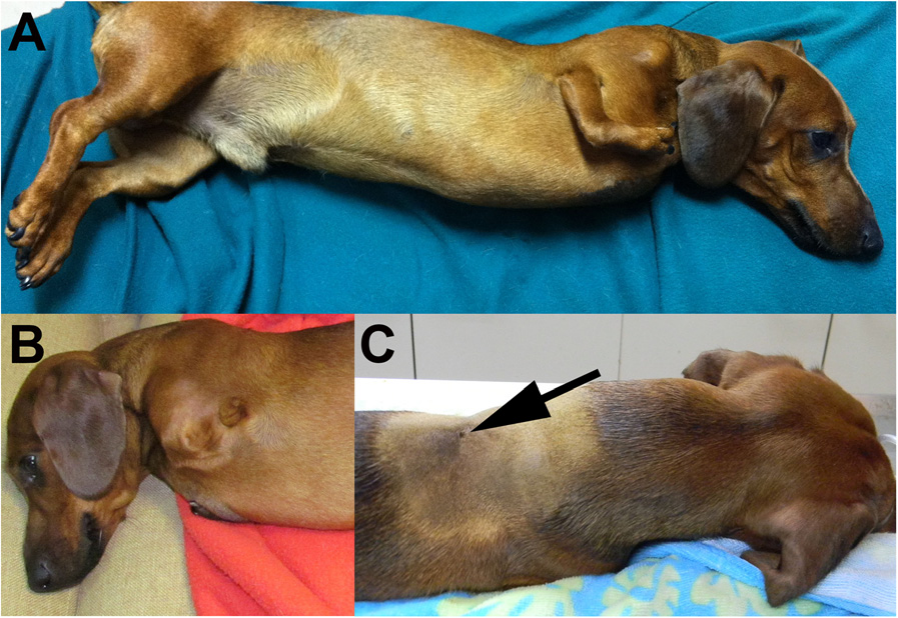
Photograph of an adult Dachshund with right thoracic limb hypoplasia (A), left thoracic limb aplasia (B), and a dermal orifice (arrow) at the T1-T2 dorsal midline (C).

**Figure 3:**
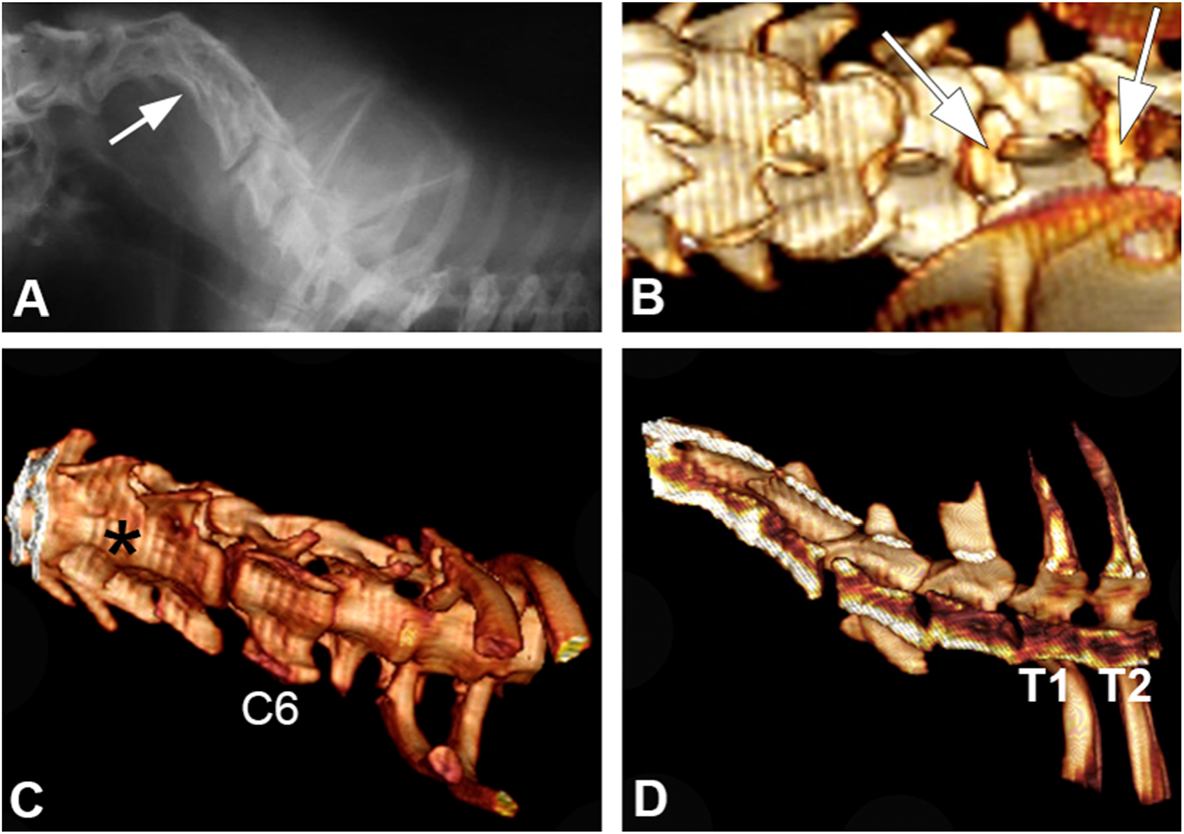
**Lateral cervical spinal radiography demonstrating fused vertebral bodies between C2 and C5 (arrow) in an adult Dachshund (A) and volume rendering 3D CT reconstruction of the dorsal cervicothoracic spine (B-D).** A widened interarcuate space was observed between C6-C7 and C7-T1 (arrows) at the dorsal cervicothoracic region **(B)**. Vertebral body fusion of C3-C5 (asterisk) is shown on ventrolateral cervical reconstruction **(C)**. A sagittal cervicothoracic view reveals T1-T2 vertebral body fusion **(D)**.

**Figure 4:**
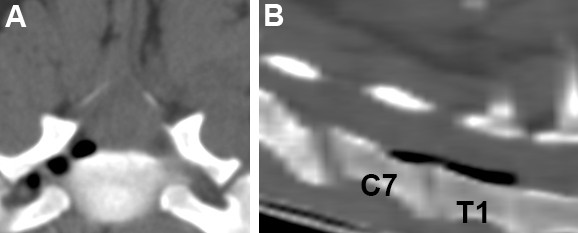
**Transverse CT image of C7/T1 reveals air within the right intervertebral foramina (A).** Reconstructed multiplanar sagittal CT images of the caudal cervical spine reveals gas within the ventral epidural space between C7-T1 **(B)**.

A 1.5-year-old, male Dachshund was presented to the Veterinary Hospital of Universidad San Sebastian, Puerto Montt, Chile, showing progressive pelvic limbs weakness and cervical pain. The owners explained that the dog was born with bilateral thoracic limbs malformations but maintained an acceptable quality of life; the dog was not receiving any medications. On physical examination, the right thoracic limb was underdeveloped, with shortened brachium (humerus), antebrachium (radio and ulna), and manus (carpus, metacarpus and digits), consistent with hypoplasia, and the left thoracic limb was absent, consistent with amelia (Figure 2). The examination also revealed cervical rigidity, hyperesthesia, muscle fasciculations, and severe cervical kyphosis. The mental status was normal and cranial nerve reflexes unremarkable, but spastic non-ambulatory paraparesis, bilateral pelvic limb proprioceptive positioning deficits and pelvic limbs extensor muscles hypertonicity were observed. The bilateral pelvic limb flexor and patellar reflexes, as well as the perineal reflex, were normal. The urinary bladder was distended, firm, and difficult to express. The thoracic limb neurologic status was impossible to evaluate because of the marked deformities. Cervical hyperesthesia was elicited on cervical flexion and extension. An orthopedic examination was not performed. A 3 mm diameter dermal orifice exuding a transparent, viscous discharge was observed at the cranial thoracic midline. The owners mentioned that the dermal discharge began 3 weeks prior to the appearance of the clinical signs. The dog’s rectal temperature (38.4°C), haematology, and serum biochemistry were within the reference ranges. Neurological findings suggested cervical (C1-C5), cervicothoracic (C6-T2) and/or thoracolumbar (T3-L3) spinal cord lesions. Differential diagnoses included inflammatory or infectious myelopathy, spinal cord developmental disorders, and spinal cord neoplasia. Cervico-thoracic radiography showed C2-C5 vertebral deformities and block vertebrae, cervical kyphosis, and T1-T2 block vertebrae (Figure 3). C2-C5 fusion and dorsal angulation produced a mildly restricted movement between C1-C2. Radiographs demonstrated humerus, radius and ulna hypoplasia in the right thoracic limb; the humerus, radius, ulna, metacarpus, and phalanges were completely absent from the left thoracic limb. Computed tomography was performed at the Veterinary Hospital at Austral University of Chile. The scans were performed using a fourth generation CT unit (Picker PQ 6000, Picker International, Cleveland, OH) under general anesthesia in sternal recumbency. The technical parameters included image acquisition with 2-mm intervals, 2-mm slice thickness, 120 kV 85 mA, and 178 mAs. Transverse, sagittal, and 3D reconstructions were obtained from the CT unit using OsiriX imaging software (OsiriX Foundation, v 3.9.2 Geneva, Switzerland). CT fistulography was performed by administering 5 ml of iohexol into the dermal orifice to visualize any potential vertebral canal communication. Computed tomography with cervical and cervicothoracic 3D reconstruction confirmed the C2-C5 and T1-T2 vertebral body fusions (Figure 3). Transverse CT images indicated the presence of round hypodense areas at the right ventrolateral epidural space of C7-T1, suggestive of epidural gas (Figure 4). Gas was also observed within the right C7-T1 intervertebral foramina. The contrast medium accumulated subcutaneously and did not reach the vertebral canal on CT fistulography. CSF analysis was not performed because the cervical vertebral malformations prevented atlanto-occipital space access to the cerebellomedullary cistern.

**Figure 5:**
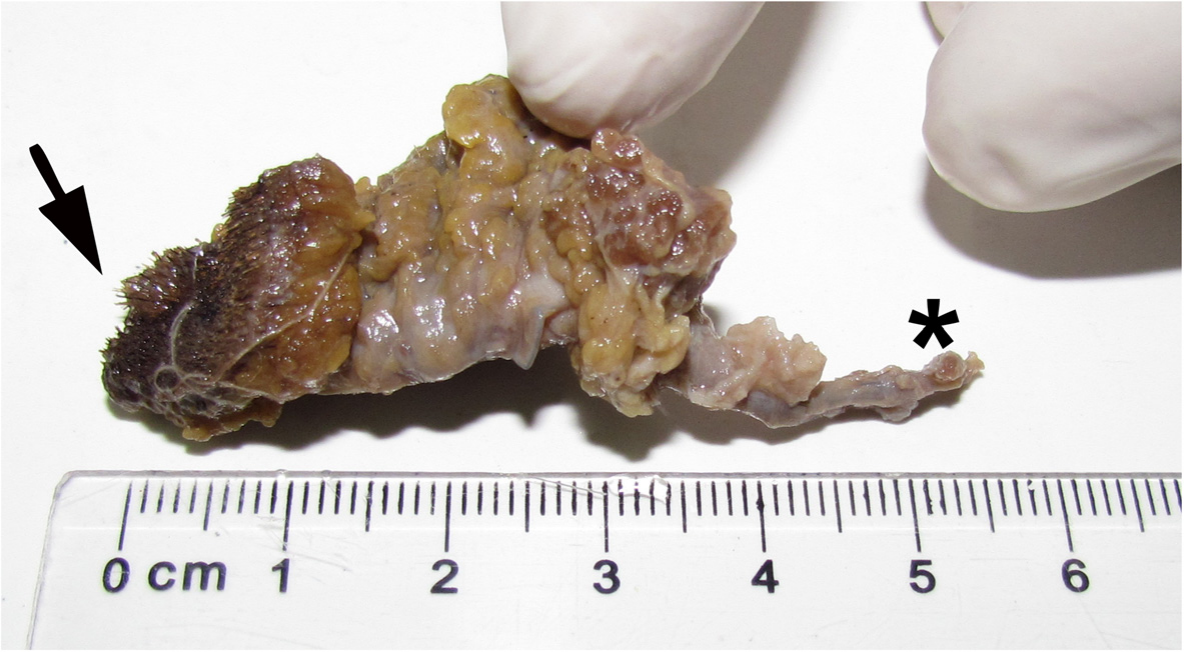
**Dermoid sinus surgically excised from a Dachshund.** The proximal epidermal base (arrow) and distal end (asterisk) are indicated.

Based on the concurrent neurological signs and diagnostic findings, meningitis was suspected, and the dermoid sinus was surgically removed. The dog was administered with 2% xylazine (1 mg/kg, IM) preanesthetically, and general anesthesia was induced with propofol (4 mg/kg, IV) and maintained with an isoflurane and oxygen mixture. To prevent pain, 2 mg/kg SC carprofen (Rimadyl, Pfizer, Argentina) was administrated 2 hours preoperatively and 2 mg/kg IV tramadol (Tramadol, Biosano, Chile) just prior to propofol induction. Intraoperative antibiotic prophylaxis was administrated using 30 mg/kg IV of sulfa and trimethoprim (Salfen, Laboratorio Chile, Chile) and 3 mg/kg IV gentamicin (Gentamicina, Laboratorio Chile, Chile). The fistulous tract was isolated from the epidermis and subcutaneous tissue by blunt dissection of the supraspinous ligament and epaxial musculature. The tract was followed and descended ventrally into the vertebral canal through the T1-T2 interarcuate space. A dorsal laminectomy was performed to completely resect the dermoid sinus. The T1-T2 interarcuate space was slightly expanded by excising a small portion of the caudal border of the vertebral lamina of T1 and the cranial border of the vertebral lamina of T2. Further dissection of the tract into the dorsal midline exposed the sinus, which extended into the dura mater through the widened T1-T2 interarcuate space. The distal end of the sinus was detached from the dura mater, the sinus was then surgically extracted (Figure 5), and the wound closed routinely. The surgery lasted 1 hour and 15 minutes and no postsurgical complications were observed.

**Figure 6:**
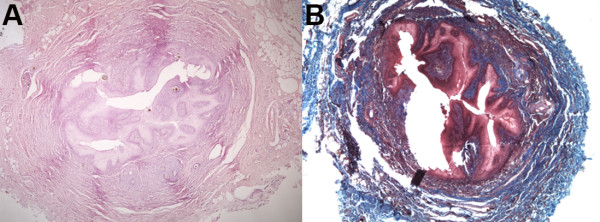
**Transverse histological photograph of the dermoid sinus.** Hematoxylin and eosin staining highlight the sinus lumen lined by squamous epithelium, sebaceous glands, hair follicles, and surrounding adipose tissue **(A)**. Masson’s trichrome stain showing abundant fibrous tissue deposition (blue) **(B)**.

The resected dermal sinus was fixed in 10% buffered formalin, processed, paraffin embedded, and sectioned at 5-μm thickness. Samples were stained with hematoxylin and eosin (H&E), Masson’s trichrome stain, and immunohistochemically targeting neuronal specific nuclear protein (NeuN) and glial fibrillary acidic protein (GFAP). Histological evaluation with H&E stain showed a dermal structure with intralumen hair follicles and sudoriparous glands, and sebaceous glands, lined with squamous epithelium and squamous debris (Figure 6). Adipose tissue lined the periphery, Masson’s trichrome stain showed that the dermoid sinus was enveloped by fibrous and collagenous tissue (Figure 6). Immunostainings for NeuN and GFAP was negative.

Postoperatively, 50 mg/kg/12 h PO sulfa and trimethoprim (Respig, Drag Pharma, Chile) for 30 days and 30 mg/kg/12 h PO metronidazole (Metronidazol L.CH., Laboratorio Chile, Chile) for 15 days were administered. Postoperative analgesia was administered with 2 mg/kg/8 h IV of tramadol (Tramadol, Biosano, Chile) and 2 mg/kg/24 h SC of carprofen for 2 days, followed by 2 mg/kg/12 h PO of tramadol (Tramal, Grünenthal, Chile) and 5 mg/kg/24 h PO of firocoxib (Previcox, Merial, Uruguay) for an additional 5 days. Several days postoperatively, a special cart was devised to provide thoracic support, which allowed the dog to walk freely. Two weeks postoperatively, pelvic limb movement and proprioceptive placing were improved, and no signs of cervical pain were observed. After 6 weeks, pelvic limb motor and sensory function were completely recovered, and no signs of cervical rigidity or cervical muscle spasms were present. The surgical site healed properly with no signs of infection.

## Discussion

The dog in the present case report suffered a congenital deformity characterized by cervical and cranial thoracic vertebral fusion (C2-C5 and T1-T2) and thoracic limbs abnormalities. These associated malformations have not been reported previously in dogs; whether these two concurrent malformations are part of a single syndrome in dogs remains unknown. Congenital cervical block vertebrae have been reported previously in dogs, but concurrent visceral or appendicular anomalies have not been observed [10]. The axial skeletal anomalies (cervical block vertebrae) described in our report are similar to those of KFS in humans, which is a congenital fusion of two or more cervical vertebrae that may be associated with other organ system anomalies, including diffuse or focal thoracic limb hypoplasia [23,25]. The dog in our study exhibited right thoracic limb hypoplasia, left thoracic limb amelia, and cervical vertebral body fusion consistent with KFS as described in humans.

Embryologically, the cervical spinal fusion results from failed mesodermal somites segmentation during the third and eighth weeks of gestation [23,25]. Limbs form during the fourth week of gestation in dogs [26]. In humans, the KFS anomaly is likely to develop during weeks 3–8 of gestation [21]. Klippel-Feil syndrome is a complex developmental disorder, and its association with several other syndromes and/or anomalies suggests a basic skeletal disorder with spontaneous or genetic etiology. Genetic mutations in *PAX1* and *MEOX1* have been implicated in the KFS pathogenesis [27,28]. These genes play important roles in somite development during embryogenesis in vertebrates. However, this syndrome likely has a heterogeneous etiology [27,28].

In dogs, cervical vertebrae fusion and block vertebrae are usually incidental radiographic findings characterized by complete or partial fusion of two or more vertebral bodies, arches, or spinous processes [29]. In dogs, cervical vertebral fusion anomalies are not clinically significant unless there is spinal instability or deformity, such as atlantoaxial instability, spinal stenosis, or angulation [24,29]. However, no previous reports in dogs have associated cervical vertebral fusion defects with appendicular skeletal anomalies.

In humans, KFS is occasionally associated with dermoid or epidermoid cysts located at the cranium posterior fossa and rarely the cervical vertebrae [30,31]. Additional neurological defects reported in KFS patients include diastematomyelia, syringomyelia, corpus callosum agnesis, meningocele, and cervical occult spina bifida, among others [32]. Dermoid sinus and KFS may arise concurrently because both processes occur during the same developmental period. The separation of the neuroectoderm from the ectoderm occurs during the weeks 3–5 of gestation [22]. Failure of this process results in cutaneous ectoderm sequestration, which forms the dermal epithelium and all skin appendages. The cutaneous ectoderm then migrates internally along with the folding of the neural tube forming a dermoid sinus [22,26]. Cervical somite segmentation also occurs during the same period [22,26]. In dogs and cats, dermoid sinus can be associated with vertebral anomalies, including block vertebrae, hemivertebrae, and partial fusion of vertebral arches (spina bifida) [6,8,12,15,18,33,34]. Spina bifida appears to be related to dermoid sinuses that extend to the vertebral canal [6,8,14,33]. In this report, the dermoid sinus was located in the widened interarcuate spaces of the first thoracic vertebrae, as observed by 3D CT reconstruction. Dermoid sinuses can also reach the spinal cord by passing through osseous defects in the vertebral laminae or vertebral spinous processes [14]. Abnormal tissue tension at the craniocervical segment secondary to abnormal flexure, vertebral fusion and cervical shortening associated with KFS has been proposed as the mechanism underlying ectodermal entrapment during neural tube closure [35,36].

In humans and dogs with type IV dermoid sinus, meningitis or meningomyelitis may be caused by the infection from the fistulous tract via an opening between the skin and the dura mater or the skin and spinal subarachnoid space [8,37,38]. CSF or purulent discharge may be observed at the dermoid sinus orifice arising from the subarachnoid space [38]. In the present report, a transparent viscous fluid discharge was observed at the dorsal midline skin orifice at T1-T2 three weeks prior to the clinical signs. Anorexia, resistance to cervical manipulation, cervical muscle fasciculations, and pelvic limbs proprioceptive deficits and weakness were considered signs of meningeal and spinal cord compromise [32]. However, CSF could not be analyzed because the vertebral malformations made CSF extraction prohibitively difficult. Surgery revealed a type IV dermoid sinus connected to the meninges, which was not observed radiographically or on CT fistulography. Clinical signs of dermoid sinus can also result from by chronic spinal cord compression by the distended type IV dermoid sinus; in the absence of a subarachnoid communication epithelial tissue and debris accumulate in the distal and compress surrounding structures [8,14]. In humans, meningitis, and intramedullary abscess have been documented in cases of spinal dermoid sinus [19,38]. Bacterial culture from a swabbed dermoid sinus in dogs has shown mixed bacterial population predominated by *Staphylococcus intermedius*[8]. Syringohydromyelia and tethering of the spinal cord has also been associated with canine dermoid sinus [6]. Contamination and extension of the inflammation, infection, and/or chronic spinal cord compression from the dermoid sinus were most likely responsible for the neurological signs observed in this report.

Dermoid sinus is considered a hereditary neural tube abnormality in Rhodesian Ridgebacks [39,40]. Their characteristic cutaneous ridge in this breed is an autosomal dominant mutation of fibroblast growth factor genes that predispose the animals to dermoid sinus [39,40]. This developmental abnormality has been reported in several other breeds, including the American Cocker Spaniel, English Cocker Spaniel, English Springer Spaniel, Boxer, Chow-Chow, Golden Retriever, Shih Tzu, Siberian Husky, Yorkshire Terrier, German Shepherd, Rottweiler, Boerboel, Great Pyrenees, Swedish Vallhund, Chinese Crested, Victorian Bulldog, and occasionally in domestic cats [6-8,11-14,16,17,41-46]. No reports of dermoid sinus in Dachshunds were found in the literature.

The spinal emphysema, also known as pneumorrhachis, observed in the present case is an uncommon event in human patients and is usually associated with traumatic events, violent coughing, intradiscal gas accumulation, surgical or anesthesic manipulations, and iatrogenic etiologies [47]. In dogs, intradiscal gas accumulation or the so-called “vacuum phenomenon” is a radiological finding that is observed in degenerative disc disease but has not been reported to produce or favor epidural gas accumulation [48]. Potentially, communication between the exterior and the vertebral canal through the dermoid sinus fistula may have introduced air into the epidural space. The epidural space has an internal pressure bellow atmospheric pressure; therefore ambient air outside of the dermal orifice presumably follows gradient pressure forces towards the vertebral canal. Alternatively, this vertebral gas accumulation may be only an incidental finding [48,49]. No previous description of epidural gas in human or animal patients diagnosed with dermoid sinus has been reported.

## Conclusions

Cervical vertebral fusion defects associated with limb malformations have not been reported in dogs and may be manifestations of a similar condition in humans known as KFS. This rare and complex bone anomaly may be associated with other congenital conditions, including dermoid sinus, and should be included on the list of canine congenital anomalies. Additionally, this is the first report of spinal dermoid sinus in a Dachshund.

## Consent

The owner provided consent for the present article.

## Competing interests

The authors declare that there were no competing interests.

## Authors’ contributions

NB and MG are the neurologists that performed the initial evaluation and neurologic examination of the dog. MG performed the CT scan evaluation and wrote the manuscript. Radiologist MM evaluated the case radiographic findings. Pathologist FV performed the histopathological and immunochemistry examination and dermoid sinus analysis. Pathology technician GA performed the immunochemistry analysis. All of the authors reviewed and approved the final manuscript.
